# Helicobacter pylori infection and its associated factors among dyspepsia patients attending Debre Tabor Comprehensive Specialized Hospital, 2020

**DOI:** 10.1371/journal.pone.0279396

**Published:** 2023-03-09

**Authors:** Tadeg Jemere, Mekdes Tilahun, Gashaw Walle, Getachew Yideg, Assefa Agegnehu, Anemut Tilahun, Edget Abebe, Natnael Moges

**Affiliations:** 1 Department of Biomedical Sciences, College of Health Sciences, Debre Tabor University, Debre Tabor, Ethiopia; 2 Department of Medical Laboratory, College of Health Sciences, Debre Tabor University, Debre Tabor, Ethiopia; 3 Department of Pediatrics and Child Health Nursing, College of Health Sciences, Debre Tabor University, Debre Tabor, Ethiopia; Nazarbayev University School of Medicine, KAZAKHSTAN

## Abstract

**Introduction:**

Helicobacter pylori is one of the most common bacterial infections of humankind that affects more than 50% of the world’s population. It has been implicated as an important agent in the pathogenesis of peptic ulcer disease and gastric cancer. Data regarding its prevalence using stool antigen test is scarce in Ethiopia. Hence, the main aim of this study is to determine the prevalence of Helicobacter pylori infection among dyspeptic patients using stool antigen test and assessing the potential risk factors.

**Methods:**

Institution based cross-sectional study was conducted on 373 dyspepsia patients. Data were collected using a pre-tested interviewer administered questionnaire. SPSS Version 23 for Windows software was used for summarization and analyses of data. Bivariate analysis was conducted to detect the association between dependent and independent variables, and all candidate variables were entered into multivariate logistic regression. Statistical significance was set at p-value <0.05.

**Result:**

More than one-third (34%) of dyspepsia patients were positive for H. pylori stool antigen test. Having greater than or equal to four children in the house [AOR = 7.5 95% CI (1.7, 33.6) *p* = 0.008)], absence of latrine for the house hold [AOR = 4.3 95% CI (1, 17.8), p = 0.043 and drinking of river water [AOR = 12.5 95% CI (1.5, 105), p = 0.021] were predictors of H-pylori infection.

**Conclusion:**

More than one-third of dyspepsia patients were positive for H-pylori infection. Overcrowding and poor hygienic conditions are the main risk factors of H-pylori infection.

## Introduction

Helicobacter pylori (H. pylori) is a Gram-negative, spiral shaped and acid-resistant bacterium which lives in the luminal surfaces of gastric epithelium [1]. It is one of the most common chronic bacterial infections of humans that affects more than 50% of the world’s population making it the most widespread infection across the globe [2]. Helicobacter pylori bacteria has been implicated as an important agent in the pathogenesis of peptic ulcer disease and considered as an essential factor for the development of gastric cancer. For this reason, the World Health Organization (WHO) has classified this bacteria as carcinogenic [3, 4]. According to global cancer observatory 2020, the prevalence of gastric cancer in Ethiopia was 3.3% [5].

Helicobacter pylori infection is asymptomatic in most cases and only 17% of individuals who tested positive for H-pylori infection will develop peptic ulcers disease which leads to dyspepsia [3]. Dyspepsia, also known as indigestion, refers to discomfort or pain that occurs in the upper abdomen, often after eating or drinking which is not a disease but a symptom. It is a common problem, affecting up to 29% of the population. Common symptoms include bloating, discomfort, feeling too full, nausea, and gas [6]. Dyspepsia is the most common illness in the Ethiopian population visiting outpatient department of health facilities, and it has also been associated with H.pylori infection [7]. Result of different studies showed that, the prevalence of H.pylori positivity among dyspepsia patients using stool antigen test varies from 23% to 65%, depending on age, geographic location, hygienic condition, life style and socioeconomic status of the populations [7–9]. The ability of H-pylori to produce large amounts of unease enzyme which hydrolyzes urea to ammonia and carbon dioxide, the presence of flagella which is important for the movement of the bacteria and persistently colonize the mucus layer of the gastric mucosa are the major contributing factors for the pathogenesis of this bacteria [7]. According to the finding of different studies, gender, occupation, poor sanitary conditions, overcrowding, and unsafe water supply sources are the factors that influence the prevalence of H. pylori infection [1, 8, 10].

Even though the serological prevalence of H. pylori infection among dyspepsia patients is known, data regarding antigenic prevalence of H. pylori among dyspepsia patients is scarce in Ethiopia. Hence, the current study was conducted with an aim to determine the prevalence of H. pylori infection among dyspeptic patients using stool antigen test which is good in detecting acute infection and distinguishing active infection from previous exposure and assessing potential risk factors.

## Methods and materials

### Study setting and period

Institution based cross-sectional study was conducted at Debre Tabor Compressive Specialized Hospital (DTCSH) in Debre Tabor town. Debre Tabor town is located in south Gondar zone, Amhara regional state, 665 kilometers northwest of Addis Ababa. DTCSH is one of the oldest hospitals in Ethiopia providing service to an estimated 2.7 million population in the catchment area. The study was conducted from August 1 to November 30, 2020

### Source and study populations

The source population were all adult dyspepsia patients who get health care at DTCSH during the study period and the study population were all adult dyspeptic patients who were requested for H. pylori stool antigen test during the study period.

### Eligibility criteria

All dyspeptic patients aged ≥18 years, who were requested for *H*. *pylori* stool antigen test at DTCSH during the study period were included. Patients who were requested for *H*.*pylori* stool antigen test but refused to give stool sample to the laboratory were excluded during the study period.

### Sample size determination and sampling technique

Single population proportion formula was used to determine sample size and the prevalence (P) 37.6% was taken from similar study in Gondar [7], with a marginal error of 5% and 95% confidence interval and 10% non-response rate. The final sample size for this study was 397. The study participants were selected by using consecutive sampling technique.

### Data collection and processing

Data on socio-demographic and behavioral characteristics of the study participants were collected using structured pre-tested interviewer administered questionnaire. Approximately 2 gram of stool sample was collected from each participant in a clean container. H. pylori stool antigen was detected by Wondfo one step H. pylori feces test kit (Guangzhou Wondfo Biotech, China) according to the manufacture`s instruction.

### Operational definition

Dyspepsia- was diagnosed as a patient having epigastric pain, abdominal pain, abdominal bloating, vomiting, early satiety, and heartburn. For this study, dyspepsia was defined as having one or more of these symptoms for 3 months or more.

### Data quality management

The reliability of the study findings was guaranteed by implementing quality control (QC) measures throughout the whole process. All materials, equipment’s and procedures were adequately controlled. Pre-tested interviewer administered questionnaire was used for data collection on socio demographic characteristics and associated factors and there was a daily follow up by the principal investigator and supervisor. To minimize bias training was given for data collectors, high emphasis was also given in designing data collection instrument, and the participants were interviewed independently so as to minimize social desirability bias.

### Data analysis

Data analysis was made by using SPSS Version 23 for Windows software. Frequencies, means and proportions were used for the descriptive analysis of data. Bivariable logistic regression was computed to test for the presence of association between H. pylori infection and independent variables and all variables were modelled in multi-variable logistic regression to identify potential risk factors using backward logistic regression. Statistical significance was set at p-value ≤ 0.05.

### Ethical consideration

Ethical clearance was obtained from the Ethics Committee of Debre Tabor University with Ethics approval number of DTU/192/2020. Informed written consent was obtained from the study participants to start data collection and the respondents were assured that their responses will remain confidential.

## Results

### Socio demographic characteristics of study participants

A total of 373 dyspepsia patients participated in this study making a response rate of 94%. From all participants, 69.7% of them were females and 51.7% were urban dwellers. Nearly half (48.3%) of study subjects were in the age range of 31–60 years followed by 18–30 years (43.2%). Nearly three-fourth of participants (73.7%) were married and 65.4% had children. From all study participants, 39.7% were farmers, 40.8% were illiterate and 74% of participants had middle income (Table 1).

**Table 1:** Socio-demographic characteristics of study participants at DTCSH, Northwest Ethiopia, 2020.

Variable		Number	Frequency (%)
Sex	Male	113	30.3
	Female	260	69.7
	18–30	161	43.2
Age	31–60	180	48.3
	>60	32	8.5
Residence	Urban	193	51.7
	Rural	180	48.3
Marital status	Single	83	22.3
	Married	275	73.7
	Other	15	4
Have children	Yes	228	61.1
	No	135	38.9
Number of Children	1	28	12.3
	2	41	18
	3	37	16.2
	≥4	122	53.5
Occupation	Employed	81	21.8
	Merchant	43	11.5
	Farmer	147	39.4
	Students	59	15.8
	Unemployed	35	9.4
	Pensioners	8	2.1
Education	Non-educated	152	40.8
	Primary school	69	18.5
	Secondary school	50	13.4
	Diploma and above	102	27.3
Income	Middle level	276	74
	Low level	97	26

### Behavioral characteristics of study participants

Among all participants, majority (96.8%) had latrine, 74.8% using pipe water for drinking and 68.9% did not eat raw vegetable. Around 98.9% of participants did not chew khat and smoke cigarette. All (100%) of respondents had a habit of hand washing before meal, after meal and after toilet but only 78.6% of participants were wash their hands with water and soap **(Table 2).**

**Table 2:** Behavioral factor of study participants at DTCSH, Northwest Ethiopia, 2020.

Variables		Number	Frequency (%)
Latrine	No	12	3.2
	Yes	361	96.8
Drinking water	Pipe water	279	74.8
	Spring water	87	23.3
	River water	7	1.9
Raw vegetable	No	257	68.9
	Rarely	91	24.4
	Always	25	6.7
Chewing khat	Rarely	4	1.1
	Never	369	98.9
Smoking Cigarette	Rarely	4	1.1
	Never	369	98.9
Drinking alcohol	Always	30	8.0
	Rarely	128	34.3
	Never	215	57.6
Wash hand before meal	Yes always	373	100.0
Wash hand after meal	Yes always	373	100.0
Wash hand after toilet	Yes always	373	100.0
Use to wash hand	Only water	76	20.4
	Water and soap	293	78.6
	Water and other ingredient	4	1.1

### Prevalence of H. pylori among dyspepsia patients

Among 373 participants, 34% of dyspepsia patients with 95% CI (29.5% to 39.4%) were positive for H. pylori stool antigen test.

### Factors associated with H-pylori infection among dyspepsia patients

From all candidate variables which were included in the backward logistic regression model, three variables were found to be statistically significant (*p*<0.05). Accordingly, number of children [having greater than or equal to four children in the house, AOR = 7.5 95% CI (1.7, 33.6) *p* = 0.008)], absence of latrine for the house hold [AOR = 4.3 95% CI (1, 17.8), p = 0.043 and drinking of river water [AOR = 12.5 95% CI (1.5, 105), p = 0.021] were predictors of H-pylori infection among dyspepsia patients (Table 3).

**Table 3:** Factors independently associated with H-pylori infection among dyspepsia patients at DTCSH, Northwest Ethiopia, 2020 (N = 373).

	H-pylori infection		OR (95% CI)			
Variables	Positive N (%)	Negative N (%)	COR (95% CI)	*p-*value	AOR (95% CI)	*p-*value
Sex Female	77(30.2)	178(69.8)	1		1	
Male	50(42.4)	68(57.6)	1.7(1.08, 2.67)	**0.022**	1.5(0.76, 3)	0.237
Number of children One	2(7.1)	26(92.9)	1		1	
Two	7(17.1)	34(82.9)	2.7(0.5, 13.9)	**0.24**	2(0.4, 10.8)	0.427
Thee	2(5.4)	35(94.6)	0.7(0.09, 5.6)	**0.77**	0.6(0.08, 4.9)	0.669
≥Four	51(41.8)	71(58.2)	9.34(2.1, 41.13)	**0.003**	7.5(1.7, 33.6)	0.008*
Presence of latrine Yes	112(31.8)	239(68.1)	1		1	
No	15(68.2)	7(31.8)	4.6(1.81, 11.53)	**0.001**	4.3(1, 17.8)	0.043*
Drinking water						
Pipe	83(30.9)	186(69.1)	1		1	
Spring	34(37)	58(63)	1.3(0.8, 2.2)	0.281	1(0.52, 2.2)	0.86
River	10(83.3)	2(16.7)	11.2(2.4, 52.3)	**0.002**	12.5(1.5, 105)	0.021*

* Statistically significant at *p-*value <0.05; COR: Crude Odds Ratio; AOR: Adjusted Odds Ratio

## Discussion

Helicobacter pylori infection is an important public health issue globally with high prevalence among developing countries [10]. It is a major pathogenic factor for gastroduodenal ulcer disease and gastric carcinoma [11]. It have been demonstrated worldwide and affect all age groups. It is estimated that 50% of the world’s population is infected with H. pylori making it the most widespread infection across the globe [2]. Its transmission has been reported to be promoted by low socio-economic status, poor quality of drinking water, overcrowding, poor personal and environmental hygiene and food contamination [1, 8].

In this study, H. pylori infection was found in 34%, of dyspepsia patients with 95% CI (29.5% to 39.4%). This is in agreement with the result of the studies conducted in Colombia (36.4%) [12], Western Uganda (29.9%) [13], Gondar (37.6%) [7], Addis Ababa (36.8%) [14] and Uganda (35.7%) [15]. However, this finding is not comparable to studies conducted in Hosaena (51.4%) [16], Butajira, 52.4% [17], Nigeria (23.5%) [9] and Egypt (64.6%) [8]. The possible reason for this discrepancy might be due to differences in socio-economic status, environmental exposure, sample size, hygienic condition of the participants and risk factors across geographic location. Although the magnitude of H. pylori in the study area is high, it was expected to be more than the observed prevalence. This might be due to the use of Proton pump inhibitors, or other drugs like antibiotics by dyspeptic patients as a treatment might have influenced the results.

There are conflicting reports on the relationship between gender of dyspepsia patients and H. pylori infection in the previous studies. Some studies show significantly higher H. pylori prevalence in males than females [7, 15, 18] and others showed a higher prevalence in females though not statistically significant [16, 19]. In this study, there was a slight difference in gender prevalence (male: 42.4% and female: 30.2%); however, this difference was not statistically significant (*p* = 0.237).

According to this study, having four or more children in the family was statistically associated with H. pylori infection, which is comparable with the results of studies conducted in Dessie [10], Egypt [8] and Cameroon [1]. However, this is not comparable with the findings of the studies done in Addis Ababa [14], and Assosa [2]. This might be due to the difference in socioeconomic status of the study population.

A significantly high infection rate was also observed in participants with the absence of latrine for the house hold (68.2%) as compared with participants who had latrine (31.8%). This is in line with the results of the study conducted in Egypt [8]. Drinking river water was also the predictor of H.pylori infection among dyspepsia patients in this study. This is in agreement with the studies conducted in Egypt [8], Yemen [18] and Pakistan [20]. This might be due to the fact that, absence of latrine and drinking river water create poor hygienic condition in the family which is a risk factor for easily transmission of H.pylori infection.

According to this study, age and marital status were not statistically associated with H. pylori infection, which is comparable with studies done in Gondar [7], and Uganda (15). Contrary to our study, a study done in Yemen (18) and China (19) showed significant association between age, marital status and H. pylori infection. In the present study, there was no statistical association between H.pylori infection with residence, family income, educational status, drinking and smoking habits.

### Strength and limitation of the study

Using a stool antigen test to assess the prevalence of H. pylori among study participants and using validated tool were the strengths of this study. However, failure to include controls and the single-center nature of this study may affect its generalizability to the entire population.

## Conclusions

More than one-third of dyspepsia patients had H. pylori infection which indicates as it is a major public health problem. Overcrowding, absence of latrine for the family and drinking river water were predictors of H. pylori infection among dyspepsia patients.

## Supporting information

S1 Appendix Amharic version questionnaire. (DOCX)

S2 Appendix English version questionnaire. (DOCX)

S1 Data (SAV)
