# Supplementary material for: Helicobacter pylori infection and its associated factors among dyspepsia patients attending Debre Tabor Comprehensive Specialized Hospital, 2020
Source: PLoS One. 2023 Mar 9;18(3):e0279396. doi: 10.1371/journal.pone.0279396 (PMC9997881; doi:10.1371/journal.pone.0279396)
Supplement: S1 Appendix — (DOCX) [file pone.0279396.s001.docx]

Appendex II: Amharic version Questionnaire

ክፍል አንድ

1. ጾታ ሀ. ወንድ ለ. ሴት
2. እድሜ----------
3. እምነትህ/ሽ ምንድን ነው?

ሀ. ሙስሊም

ለ. ኦርቶዶክስ

ሐ. ጼንጤ

መ. ሌላ እምነት

1. የትዳር ሁኔታ ምን ይመስላል?

ሀ. ያላገባ/ች

ለ. ያገባ/ች

ሐ. የፈታ/ች

መ. ጋለሞታ

1. ልጅ አለህ/ሽ? ሀ. አለገኝ ለ. የለኝም
2. ስንት ልጆች አሉህ/ሽ?

ሀ. አንድ

ለ. ሁለት

ሐ. ሶስት

መ. አራት እና ከአራት በላይ

1. የመኖሪያ ቦታህ/ሽ የት ነው? ሀ. ከተማ ለ. ገጠር
2. የትምህርት ደረጃህ/ሽ ምንድን ነዉ?

ሀ. ያልተማረ

ለ. የመጀመሪያ ደረጃ

ሐ. ሁለተኛ ደረጃ

መ. ዲፕሎማ ከዛ በላይ

1. የገቢ ምንጭህ/ሽ ምን ይመስላል?

ሀ. ከፍተኛ

ለ. መካከለኛ

ሐ. ዝቅተኛ

1. የምትሰራው ሰራ ምንድን ነው?

ሀ. የመንግስት ስራ

ለ. ግብረ ሰናይ ድርጅት

ሐ. አርሶ አደር

መ. ነጋዴ

1. ሽንት ቤት አለህ/ሽ? ሀ. አለን ለ. የለንም
2. እጅህን/ሸን ከምግብ በፊት ትታጠባለህ/ሽ?

ሀ. አልታጠብም

ለ. አንዳዴ እታጠባለሁ

ሐ. ሁሌም እታጠባለሁ

1. እጅህን/ሸን ከምግብ በኍላ ትታጠባለህ/ሽ?

ሀ. አልታጠብም

ለ. አንዳዴ እታጠባለሁ

ሐ. ሁሌም እታጠባለሁ

1. **እጅህን/ሽን ከሽንት ቤት መልስ ትታጠባለህ/ቤለሽ?**

**ሀ. አልታጠብም**

**ለ. አንዳዴ እታጠባለሁ**

**ሐ. ሁሌም እታጠባለሁ**

1. የመጠጥ ውሀህ/ሽ ምንድን ነው?

ሀ. የቧምቧ ውሀ

ለ. የምንጭ ውሀ

ሐ. የታሸገ ውሀ

መ. የወንዝ ውሀ

1. እጅህን/ሽን ለመታጠብ ምትጠቀሚው/መው ምንድን ነው?

ሀ. ውሀ ብቻ

ለ. ውሀ እና ሳሙና

ሐ. ውሀ እና ሌላ ነገር

1. አትክልት ሳይሰራ ትመገባለህ/ሽ?

ሀ. አልጠቀምም

ለ. አንዳንዴ እጠቀማለሁ

ሐ. እጠቀማለሁ

1. ጫት ቅመህ ታውቃለህ/ሽ?

ሀ. አልጠቀምም

ለ. አንዳንዴ እጠቀማለሁ

ሐ. እጠቀማለሁ

1. ሲጋራ አጭሰህ/ሽ ታውቃለህ/ቂያለሽ?

ሀ. አልጠቀምም

ለ. አንዳንዴ እጠቀማለሁ

ሐ. እጠቀማለሁ

1. አልኮል ጠጥተህ/ሽ ታውቃለህ/ቂያለሽ?

ሀ. አልጠቀምም

ለ. አንዳንዴ እጠቀማለሁ

ሐ. እጠቀማለሁ

1. ነፍሰጡር ነሽ? ሀ. አወ ለ. አይደለሁም
2. የምግብ ፍላጎትሽ ቀንሷል?

ሀ. አወ ለ. ትንሽ ትንሽ እመገባለሁ ሐ. መመገብ እችላለሁ

23. ያስታውክሻል? ሀ. አወ ለ. የለም

24. የኤች ፓይሎሬ ምርመራ ውጤት

ሀ. ፖዘቲቭ

ለ. ነጌቲቭ
