# Supplementary material for: Helicobacter pylori infection and its associated factors among dyspepsia patients attending Debre Tabor Comprehensive Specialized Hospital, 2020
Source: PLoS One. 2023 Mar 9;18(3):e0279396. doi: 10.1371/journal.pone.0279396 (PMC9997881; doi:10.1371/journal.pone.0279396)
Supplement: S2 Appendix — (DOCX) [file pone.0279396.s002.docx]

## Appendix I: English Version Questionnaire

Part 1:

1. Sex
2. Male
3. Female
4. Age----------------
5. Religion
6. Orthodox
7. Muslim
8. Protestant
9. others
10. Marital status
11. Single
12. Married
13. Divorced
14. Window
15. Do you have children?
16. Yes
17. No

If the answer is yes go to question number 6

1. Number of children
2. One
3. Two
4. Three
5. Greater than four
6. Residence
7. Urban
8. Rural
9. Educational level
10. Non-educated
11. Primary school
12. Secondary school
13. Diploma and above
14. Monthly income
15. High level
16. Middle level
17. Low level
18. Occupation
19. Governmental office
20. Non-governmental office
21. Merchant
22. Farmer
23. others

Part II Behavioral factors towards *H.pylori* infection

1. Do you have latrine in your home?
2. No
3. Yes
4. Do you wash your hands before meal?
5. No
6. Rarely
7. Yes, always
8. Do you wash your hands after meal?
9. No
10. Rarely
11. Yes, always
12. Do you wash your hands after toilet?
13. No
14. Rarely
15. Yes, always
16. What is your source of drinking water?
17. Pipe water
18. Spring water
19. Packed water
20. River water
21. What do you use to wash your hands?
22. Water only
23. Water and soap
24. Water and other ingredients
25. Have you ever eating raw vegetables?
26. No
27. Rarely
28. always
29. Have you ever chewing chat?
30. Always
31. Rarely
32. Never
33. Have you ever smoking cigarette?
34. Always
35. Rarely
36. Never
37. Have you ever drinking alcohol?
38. Always
39. Rarely
40. Never

Part III Physiological factors towards *H.pylori* infection

1. Are you pregnant?
2. Yes
3. No

If the answer is yes for question number 23 go to question 24

1. Do you have loss of appetite?
2. No, I am able to eat
3. Partially, I can eat
4. Completely, I can’t able to eat
5. Does the pregnant woman vomits?
6. Yes
7. No

Part IV pathological factors towards *H.pylori* infection

1. What is *H.pylori* result for the client?
2. Positive B. Negative
